# Activation of Muscarinic Acetylcholine Receptor Subtype 4 Is Essential for Cholinergic Stimulation of Gastric Acid Secretion: Relation to D Cell/Somatostatin

**DOI:** 10.3389/fphar.2016.00278

**Published:** 2016-08-30

**Authors:** Koji Takeuchi, Takuya Endoh, Shusaku Hayashi, Takeshi Aihara

**Affiliations:** ^1^Division of Pathological Sciences, Department of Pharmacology and Experimental Therapeutics, Kyoto Pharmaceutical UniversityKyoto, Japan; ^2^General Incorporated Association, Kyoto Research Center for Gastrointestinal DiseasesKyoto, Japan

**Keywords:** acid secretion, isolated mouse stomach, carbachol, somatostatin, knockout mouse, muscarinic receptor subtypes, SST2 receptor

## Abstract

**Background/Aim**: Muscarinic acetylcholine receptors exist in five subtypes (M_1_∼M_5_), and they are widely expressed in various tissues to mediate diverse autonomic functions, including gastric secretion. In the present study, we demonstrated, using M1∼M5 KO mice, the importance of M4 receptors in carbachol (CCh) stimulation of acid secretion and investigated how the secretion is modulated by the activation of M4 receptors.

**Methods:** C57BL/6J mice of wild-type (WT) and M1–M5 KO were used. Under urethane anesthesia, acid secretion was measured in the stomach equipped with an acute fistula. CCh (30 μg/kg) was given subcutaneously (s.c.) to stimulate acid secretion. Atropine or octreotide (a somatostatin analog) was given s.c. 20 min before the administration of CCh. CYN154806 (a somatostatin SST2 receptor antagonist) was given i.p. 20 min before the administration of octreotide or CCh.

**Results:** CCh caused an increase of acid secretion in WT mice, and the effect was totally inhibited by prior administration of atropine. The effect of CCh was similarly observed in the animals lacking M1, M2 or M5 receptors but significantly decreased in M3 or M4 KO mice. CYN154806, the SST2 receptor antagonist, dose-dependently and significantly reversed the decreased acid response to CCh in M4 but not M3 KO mice. Octreotide, the somatostatin analog, inhibited the secretion of acid under CCh-stimulated conditions in WT mice. The immunohistochemical study showed the localization of M_4_ receptors on D cells in the stomach. Serum somatostatin levels in M4 KO mice were higher than WT mice under basal conditions, while those in WT mice were significantly decreased in response to CCh.

**Conclusions:** These results suggest that under cholinergic stimulation the acid secretion is directly mediated by M3 receptors and indirectly modified by M4 receptors. It is assumed that the activation of M4 receptors inhibits the release of somatostatin from D cells and minimizes the acid inhibitory effect of somatostatin through SST2 receptors, resulting in enhancement of the acid response mediated by M3 receptors on parietal cells.

## Introduction

The mechanisms that govern gastric acid secretion involve neuro-humoral factors, including histamine, gastrin, and acetylcholine (ACh; Soll, 1994; Hersey and Sachs, 1995; Chen et al., 2004). The action of histamine is mediated intracellularly by 3′, 5′-cyclic adenosine monophosphate (cAMP), while those of gastrin and ACh are mediated by an increase of intracellular Ca^2+^ (Thurston et al., 1979). The stimulatory actions of histamine and gastrin are mediated by the activation of histamine H_2_ and cholecystokinin (CCK)-2 receptors, respectively (Brimblecombe et al., 1978; Hersey and Sachs, 1995; Chen et al., 2004), and that of ACh is caused by the activation of muscarinic acetylcholine receptors (mAChRs; Soll, 1994; Caulfield and Birdsall, 1998).

The mAChRs consist of five subtypes (M1–M5) and are widely expressed in many peripheral organs as well as central nervous system to mediate diverse autonomic functions, including acid, pepsin and mucus as well as ${\mathrm{HCO}}_{3}^{–}$ secretions (Kajimura et al., 1992; Helander et al., 1996; Caulfield and Birdsall, 1998; Matsui et al., 2000, 2004; Tobin et al., 2009). These receptors are G protein-coupled receptors; M1, M3, and M5 receptors are coupled to Gq protein, while M2 and M4 receptors are coupled to Gi protein (Caulfield and Birdsall, 1998; Matsui et al., 2000). It is generally accepted that the acid stimulatory action of carbachol (CCh), a muscarinic agonist, is mediated by the activation of M1 and M3 receptors (Nakamura et al., 1985; Pfeiffer et al., 1990). However, Aihara et al. (2005) examined the involvement of M1, M3, and M5 receptors in cholinergic regulation of acid secretion using muscarinic receptor knockout (KO) mice and found that CCh-stimulated acid secretion is mediated by mainly M3 and partially M5 but not M1 receptors. In addition, we recently found that CCh-induced duodenal ${\mathrm{HCO}}_{3}^{–}$ secretion was markedly decreased in M4 KO mice, suggesting the involvement of M4 receptors in the cholinergic stimulation of ${\mathrm{HCO}}_{3}^{–}$ secretion (Takeuchi et al., 2015). However, no information is currently available on the role of M4 receptors in the cholinergic regulation of gastric acid secretion.

Somatostatin, a peptide hormone, regulates many physiological functions in the gastrointestinal tract (Lucey and Yamada, 1989). This peptide is produced and secreted from D cells and inhibits acid secretion through a tonic inhibitory effect on both parietal and enterochromaffin-like cells via the activation of G protein-coupled SST2 receptors (Warhurst et al., 1996; Patel, 1997; Piqueras and Martínez, 2004). Chiba and Yamada (1990) reported that CCh inhibited both basal and pentagastrin-stimulated somatostatin secretion in the isolated canine D cells in a Gi protein/cAMP-dependent manner. We reported the involvement of somatostatin in the regulatory mechanism of the CCh-stimulated ${\mathrm{HCO}}_{3}^{–}$ secretion (Takeuchi et al., 2015). It is thus possible that endogenous somatostatin may also be involved in the mechanism of gastric acid secretion in response to cholinergic stimulation, although no study has tested this hypothesis.

In the present study, we examined the effects of CCh on gastric acid secretion in wild-type (WT) and mAChR KO mice lacking M1–M5 receptors, and investigated the involvement of M4 receptors in the stimulatory action of CCh. We demonstrated the importance of M4 receptors in the cholinergic stimulation of gastric acid secretion and showed how this secretion can be modulated by the activation of M4 receptors, particularly focusing on the relation to D cell/somatostatin.

## Materials and Methods

### Animals

Age-matched male C57BL/6J mice, weighing 25∼30 g of WT and those lacking M1, M2, M3, M4, or M5 receptor, were used. The generation and characterization of each subtype of mAChR KO mouse strain has been previously described by others (Matsui et al., 2000; Ohno-Shosaku et al., 2003; Nakamura et al., 2004). Animals were housed in plastic cages with hardwood chips in an air-conditioned room (25°C), and were given standard dry pellets, CA-1 (CLEA Japan, Tokyo, Japan) and water *ad libitum*. All experimental procedures used were carried out in accordance with the Helsinki Declaration and have been approved by the Committee for Animal Experimentation established by Kyoto Pharmaceutical University.

### Determination of Gastric Acid Secretion

Acid secretion was measured in acute fistula stomachs of both WT and M1–M5 KO mice according to a previously published method (Niida et al., 1991; Kitamura et al., 1999). Under urethane anesthesia [1.25 g/kg, intraperitoneally (i.p.)], the trachea was cannulated to ensure a patent airway, and the body temperature was maintained at 36 ± 1°C using a heating lamp. Then, the abdomen was incised, both the stomach and duodenum were exposed, and the cardiac portion was ligated without interfering with vagus nerves. An acute fistula (inside diameter, 2 mm) made with a polyethylene tube was inserted into the stomach from a small incision made in the duodenum and held in place by a ligature around the pylorus. At the beginning of each experiment, the stomach was rinsed several times with physiological saline (154 mM NaCl) and filled with 0.4 ml of saline for 20 min for determination of the basal secretion. Then, the stomach was instilled with 0.4 ml of saline, and the solution was changed every 20 min. The collected samples were titrated to pH 7.0 against 2 mM NaOH using an autoburette (Comitite-8; Hiranuma, Tokyo, Japan). Gastric acid secretion was stimulated by CCh given subcutaneously (s.c.) in a dose of 30 μg/kg in both WT and KO mice lacking M1–M5 receptors. In WT mice, atropine (0.1 and 0.3 mg/kg) or octreotide (a somatostatin analog: 20 μg/kg) was given s.c. 20 min before the administration of CCh. In some cases CYN154806 (a somatostatin SST2 receptor antagonist: 0.1 mg/kg; Feniuk et al., 2000) was given i.p. 20 min before the administration of octreotide (20 μg/kg) in WT mice or the administration of CCh (30 μg/kg) in M4 KO mice. Control animals received saline or vehicle in place of the active agent. The doses of atropine, octreotide or CYN154806 were selected in order to induce the respective pharmacological actions according to the findings of previously published studies (Aihara et al., 2005; Xie et al., 2005; Terashima et al., 2009).

### Analyses for Gene Expression of mRNAs of mAChR Subtypes in Mouse Stomachs

Whole stomachs were collected from both WT mice and those lacking M1–M5 receptors, and immediately frozen in liquid nitrogen and stored at -80°C until use. Total RNA was extracted from tissue samples using Sepasol RNA I (Nacalai Tesque, Kyoto, Japan). Total RNA was reverse-transcribed with a first strand cDNA synthesis kit (ReverTra Ace alpha, TOYOBO, Osaka, Japan; Amagase et al., 2010). The sequences of the sense and antisense primers for mouse M1–M5 receptors and GAPDH, and the sizes of the expected RT-PCR products are shown in **Table 1**. An aliquot of the RT reaction product served as a template in 35 cycles of PCR with 0.5 min of denaturation at 95°C and 1 min of extension at 68°C using the Advantage 2 polymerase mixture (CLONTECH, Mountain View, CA, USA) in a thermal cycler (PC-806, ASTEC, Fukuoka, Japan; Hayashi et al., 2014). A portion of the PCR mixture was electrophoresed in 1.5% agarose gel in Tris-acetic acid-EDTA buffer (40 mM Tris, 20 mM acetic acid, and 2 mM EDTA; pH 8.1), and the gel was stained with ethidium bromide and photographed (Bio Doc-It Imaging System; UVP, Upland, CA, USA). Images were analyzed with the Image J (version 1.39), and the semi-quantitative measurement of mRNA expression was presented as a ratio compared with GAPDH (Nakamori et al., 2010; Hayashi et al., 2014).

**Table 1:** Sequences of sense and antisense primers for mouse M1–M5 receptors.

	Sequences	PCR products
Ml sense antisense	5′-GCAGCAGCTCAGAGAGGTCACAG-3′ 5′-GATGAAGGCCAGCAGGATGG-3	413 bp
M2 sense antisense	5′-GCGGATCCTGTGGCCAACCAAGAC-3′ 5′-CGAATTCACGATTTGCGGGCTA-3′	441 bp
M3 sense antisense	5′-AAGGCACCAAACGCTCATCT-3′ 5′-GCAAACCTCTTAGCCAGCGT-3′	511 bp
M4 sense	5′-AGCCGCAGCCGTGTTCACAA-3′	345 bp
Antisense	5′-TGGGTTGAGGGTTCGTGGCT-3′	
M5 sense	5′-GTCTCCGTCATGACCATACTCTA-3′	230 bp
Antisense	5′-CCCGTTGTTGAGGTGCTTCTAC-3′	
GAPDH sense	5′-GAACGGGAAGCTCACTGGCATGGC-3′	191 bp
antisense	5′-TGAGGTCCACCACCCTGTTGCTG-3′	

### Immunohistological Study

Expressions of mAChR M4 receptor and somatostatin were immunohistochemically examined in the gastric mucosa of WT or M4 KT mice. The stomachs were excised, rinsed with ice-cold PBS, and embedded in O.C.T. compound (Tissue-Tek, Sakura, Tokyo, Japan) iced with liquid CO_2_. Frozen samples were sectioned at a thickness of 10 μm at -20°C using a cryostat microtome (Leica Biosystems CM1510, Nussloch, Germany). The sections were exposed to 3% bovine serum albumin solution for 1 h to reduce the non-specific binding of anti-sera. The sections were exposed to each primary antibody for 16 h at 4°C, and incubated with the appropriate secondary antibody for 2 h at a room temperature. The sections were mounted with VECTASHIELD mounting medium, including 4,6-diamidino-2- phenylindole (Vector Laboratories, Peterborough, UK). The preparations were observed using a fluorescence microscope (Olympus BX51, Tokyo, Japan) and photographed using an Olympus digital camera. The following primary antibodies were used: rabbit anti-mAChR M4 and goat anti-Somatostatin (Santa Cruz Biotechnology, Santa Cruz, CA, USA). Alexa Fluor 488 conjugated donkey anti-rabbit IgG and Alexa Fluor 546 conjugated donkey anti-goat IgG (Molecular Probes, Eugene, OR, USA) were used as secondary antibodies.

### Determination of Serum Somatostatin Levels

Serum levels of somatostatin were measured in both WT and M4 KO mice before and after the s.c. administration of CCh (30 μg/kg), according to a previously published paper (Terashima et al., 2009). Thirty minutes after each treatment, blood was collected from the descending aorta. Then, blood samples were centrifuged at 6000 g for 15 min, and the supernatant of each sample was frozen at 20°C until the measurement of somatostatin. The concentration of somatostatin was measured with a somatostatin immunoassay kit (Peninsula Laboratories, Inc., San Carlos, CA, USA).

### Preparation of Drugs

Drugs used were urethane (Tokyo Kasei, Tokyo, Japan), carbamylcholine chloride [carbachol: CCh], octreotide, CYN154806 (Sigma-Aldrich, St. Louis, MO, USA), and atropine sulfate (Nacalai tesque, Kyoto, Japan). CCh and atropine were dissolved in saline, while octreotide and CYN154806 were dissolved in dimethyl sulfoxide (DMSO: Wako, Osaka, Japan) and diluted with distilled water to desired concentrations. Each agent was prepared immediately before use and administered as a single injection s.c. or i.p. in a volume of 1 ml per 100 g body weight.

### Statistical Analysis

Data are presented as means ± SE. Differences between two groups were evaluated with the Student’s *t*-test. Differences between multiple groups were evaluated with analysis of variance followed, when necessary, by a Dunnett’s multiple comparison test. Values of *P* < 0.05 were considered statistically significant.

## Results

### Effect of CCh on Gastric Acid Secretion in WT Mice

**FIGURE 1:**
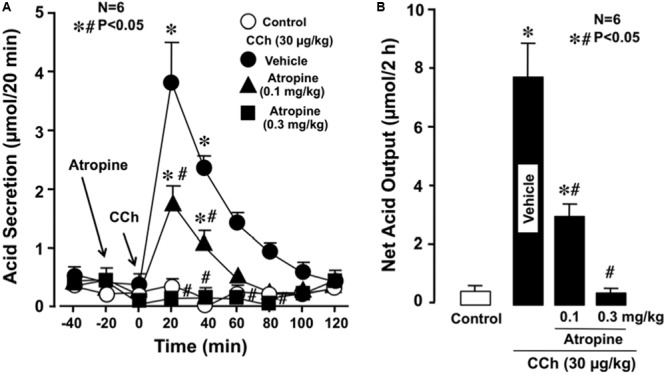
**Effects of atropine on CCh-stimulated acid secretion in the stomach of WT mice.** CCh (30 μg/kg) was administered s.c. as a single injection. Atropine (0.1 and 0.3 mg/kg) was given s.c. 20 min before the administration of CCh. **(A)** Data are presented as the mean ± SE of values determined every 20 min from six mice. **(B)** Shows the total net acid output for 2 h after the administration of CCh, and the data are presented as the mean ± SE from six mice. Significant difference at *P* < 0.05; ^∗^ from control; ^#^ from Vehicle.

Under urethane anesthesia the stomachs of WT mice spontaneously secreted acid secretion in almost negligible amount of less than 0.1–0.2 μmol/20 min. Subcutaneously administered CCh (30 μg/kg) significantly stimulated acid secretion; the acid secretion reached a peak value of 3.8 ± 0.5 μmol/20 min 20 min after the administration, followed by a gradual decrease to near basal levels 100 min later (**Figure 1A**). Pretreatment of the animals with atropine (0.1 and 0.3 mg/kg, s.c.) dose-dependently inhibited the increase of acid secretion in response to CCh, and the net acid output at 0.1 and 0.3 mg/kg was 2.8 ± 0.3 and 0.3 ± 0.1 μmol/2 h, respectively, both of which were significantly lower than that (7.9 ± 1.3 μmol/2 h) of control mice given CCh plus vehicle (**Figures 1A,B**).

### Effect of CCh on Gastric Acid Secretion in mAChR KO Mice

Since the acid stimulatory action of CCh was almost completely inhibited by atropine, the non-selective antagonist of mAChR, these results confirmed that CCh stimulated gastric acid secretion via the activation of mAChRs. We then investigated the subtype(s) of mAChRs involved in the regulation of gastric acid secretion, we examined the effects of CCh on acid secretion in the stomachs of mAChR KO mice lacking M1, M2, M3, M4, or M5 receptors.

**FIGURE 2:**
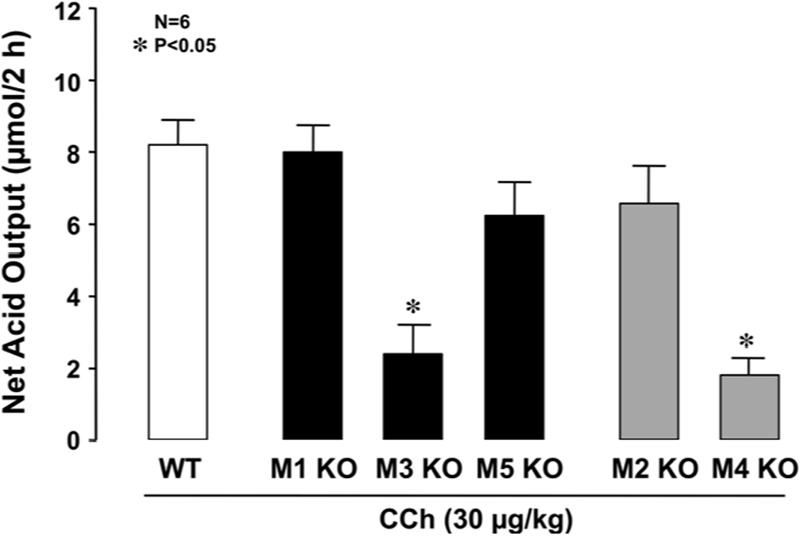
**Effects of CCh on acid secretion in the stomachs of WT mice and those of KO animals lacking M1–M5 receptors.** CCh (30 μg/kg) was administered s.c. as a single injection. Data show the total net acid output for 2 h after the administration of CCh and are presented as the mean ± SE from six mice. ^∗^Significant difference from WT at *p* < 0.05.

**FIGURE 3:**
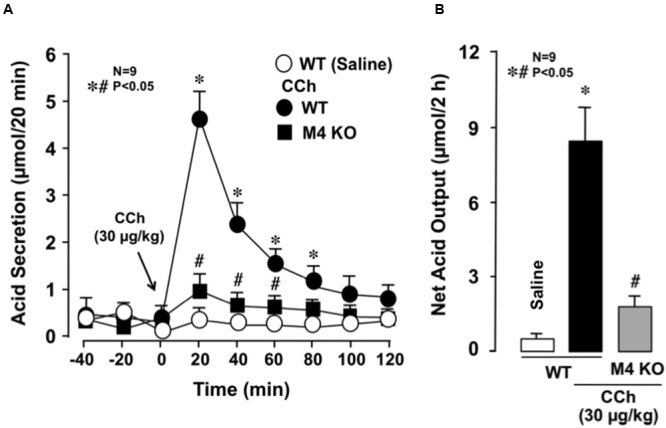
**Effects of CCh on acid secretion in the stomachs of WT and M4 KO mice.** CCh (30 μg/kg) was administered s.c. as a single injection. **(A)** The data are presented as the mean ± SE of values determined every 20 min from nine mice. **(B)** Shows the total net acid output for 2 h after the administration of CCh, and the data are presented as the mean ± SE from nine mice. Significant difference at *p* < 0.05; ^∗^ from Saline in WT; ^#^ from CCh in WT.

The stomachs of both WT and various mAChR KO mice consistently secreted 0.1∼0.3 μmol/20 min of H^+^ as basal secretion, and no significant difference was observed in the basal rates of the stomachs of these animals (data not shown). The stimulatory action of CCh (30 μg/kg, s.c.) was similar between mAChR KO mice lacking M1 and M2 receptors and WT mice, with the net acid outputs being 8.0 ± 0.8 μmol/2 h and 6.8 ± 0.9 μmol/2 h, respectively, which were almost equivalent to that (8.1 ± 0.7 μmol/2 h) in WT mice (**Figure 2**). Although a slight decrease in the acid response to CCh was observed in M5 KO mice, the net acid output (6.4 ± 0.7 μmol/2 h) was not statistically significant from that of WT mice. In contrast, the acid response to CCh was significantly decreased in M3 and M4 KO mice, with the net acid outputs being 2.3 ± 0.9 μmol/2 h and 2.1 ± 0.5 μmol/2 h, respectively. As shown in **Figure 3A**, CCh-stimulated acid secretion was markedly decreased in M4 KO mice. In WT mice the secretion of acid reached a peak value of 4.7 ± 0.7 μmol/20 min with the net acid output being 8.5 ± 1.2 μmol/2 h, while in M4 KO mice the peak value was 1.0 ± 0.2 μmol/20 min with the net acid output being 1.8 ± 0.3 μmol/2 h, which was significantly lower than that in WT mice (**Figure 3B**).

### Effects of Somatostatin on Gastric Acid Secretion in WT Mice

Somatostatin has been shown to inhibit secretory and motor functions in the gastrointestinal tract and antagonizes the actions of several hormones (Lucey and Yamada, 1989; Piqueras and Martínez, 2004; Takeuchi et al., 2015). Since the release of somatostatin from D cells is known to be mediated by an increase in cAMP (Lucey and Yamada, 1989), and the activation of M4 receptors is coupled with Gi protein to inhibit adenylate cyclase (Caulfield and Birdsall, 1998; Tobin et al., 2009), it is possible that the decreased acid response observed in M4 KO mice may be associated with changes in somatostatin secretion. Therefore, we examined the effects of octreotide, an analog of somatostatin, on gastric acid secretion in WT mice under CCh-stimulated conditions.

**FIGURE 4:**
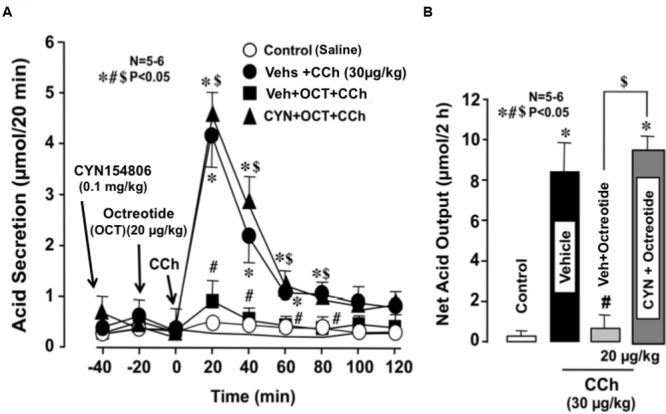
**Effects of octreotide on CCh-stimulated acid secretion in the stomachs of WT mice.** CCh (30 μg/kg) was administered s.c. as a single injection. Octreotide (20 μg/kg), an analog of somatostatin-14, was administered s.c. 20 min before the administration of CCh, while CYN154806 (0.1 mg/kg), a SST2 antagonist, was given i.p. 20 min before the administration of octreotide. **(A)** The data are presented as the mean ± SE of values determined every 20 min from 5 to 6 mice. **(B)** Shows the total net acid output for 2 h after the administration of CCh, and the data are presented as the mean ± SE from 5 to 6 mice. Significant difference at *P* < 0.05; ^∗^ from Control (saline); ^#^ from Vehicle + CCh; $ from Vehicle + Octreotide + CCh.

The basal secretion of acid in WT mice was very scanty with less than 0.1∼2 μmol/20 min and did not show significant changes following the s.c. administration of octreotide (20 μg/kg). However, the gastric acid response induced by CCh (30 μg/kg) was markedly reduced by the prior administration of octreotide; the net acid output was 0.7 ± 0.6 μmol/2 h, which was highly significantly different from that (8.5 ± 1.9 μmol/2 h) of WT mice (**Figures 4A,B**). This inhibitory effect of octreotide was completely abrogated when CYN154806 (0.1 mg/kg), a SST2 antagonist, was given 20 min before the administration of octreotide; the net acid output in response to CCh was 9.5 ± 0.8 μmol/2 h, which was almost equivalent to that of WT mice.

### Effects of the Somatostatin SST2 Receptor Antagonist CYN154806 on Gastric Acid Response to CCh in M4 KO Mice

We demonstrated that octreotide, an exogenous somatostatin analog, significantly inhibited CCh-stimulated secretion of acid in the stomachs of WT mice. Since it has been shown that CCh inhibited both basal and pentagastrin-stimulated somatostatin secretion in rats (Chiba and Yamada, 1990), there may be some interaction between somatostatin secretion and CCh-stimulated acid secretion. Then, to investigate the involvement of endogenous somatostatin in the decreased acid response to CCh in M4 KO mice, we examined the effects of CYN154806, a SST2 receptor antagonist, on the CCh-stimulated acid secretion in M4 KO mice.

**FIGURE 5:**
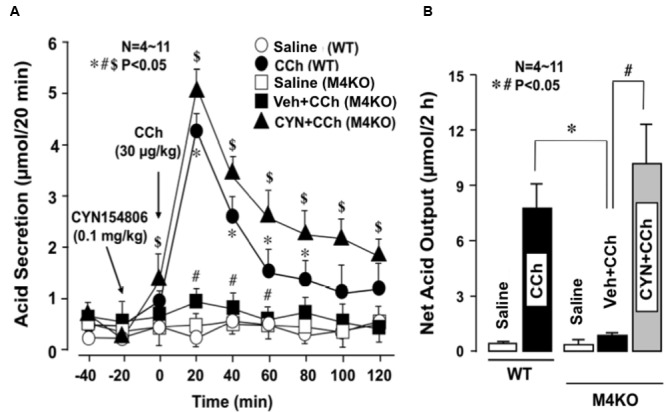
**Effects of CCh on acid secretion in the stomachs of WT and M4 KO mice, with or without the pretreatment of CYN154806.** CCh (30 μg/kg) was administered s.c. as a single injection. CYN154806 (0.1 mg/kg) was administered i.p. 20 min before the administration of CCh. **(A)** The data are presented as the mean ± SE of values determined every 20 min from 4 to 11 mice. Significant difference at *p* < 0.05, ^∗^ from Saline (WT), ^#^ from CCh (WT), $ from Vehicle + CCh (M4KO). **(B)** Shows the total net acid output for 2 h after the administration of CCh, and the data are presented as the mean ± SE of values determined every 10 min from 4 to 11 mice. Significant difference at *p* < 0.05, ^∗^ from CCh (WT), ^#^ from Vehicle + CCh (M4 KO).

Subcutaneously administered CCh (30 μg/kg) markedly increased acid secretion in the stomachs of WT mice, the peak value of acid secretion was 4.2 ± 0.2 μmol/20 min and the net acid output was 8.2 ± 1.3 μmol/2 h (**Figures 5A,B**). By contrast, CCh did not increase acid secretion in the stomachs of M4 KO mice; the peak value of acid secretion was 0.9 ± 0.1 μmol/20 min, while the net acid output was 1.0 ± 0.8 μmol/2 h, which was almost equivalent to that in the stomachs of M4 KO treated with saline. However, when M4 KO mice were pretreated i.p. with CYN154806 (0.1 mg/kg), the administration of CCh potently increased acid secretion; the peak value was 5.1 ± 0.3 μmol/20 min, while the net acid output was 10.1 ± 2.5 μmol/2 h, which was significantly greater than that of M4 KO mice without pretreatment of CYN154806. The decreased acid response to CCh in M3 KO mice was not affected by CYN154806 (data not shown).

### Immunostaining of Somatostatin and M4 Receptors in Stomach of WT and M4 KO Mice

**FIGURE 6:**
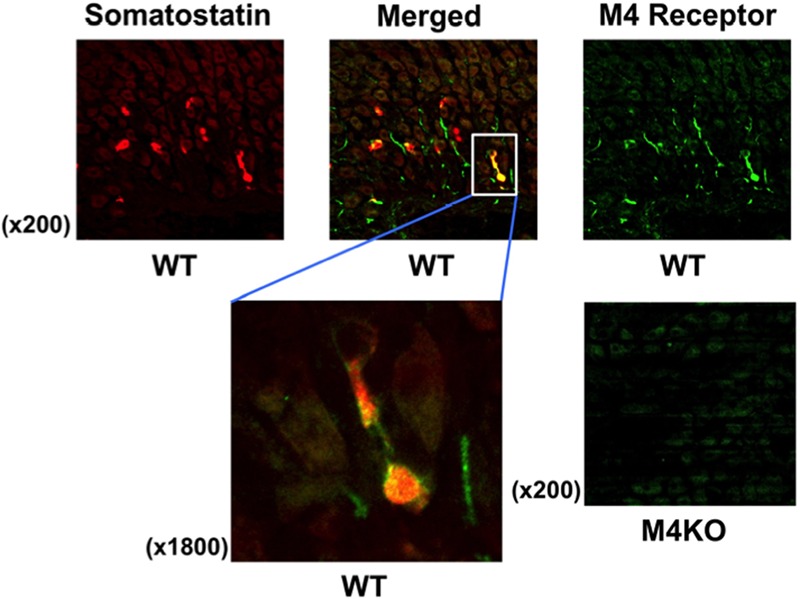
**Fluorescence immunochemical staining of the gastric mucosa with anti-somatostatin and anti-M4 receptor antibodies in WT or M4 KO mice.** Somatostatin was stained red, while M4 receptor was stained green. The left of the lower panels shows higher magnification to indicate the co-expression of M4 receptors with somatostatin on D cells in a WT mouse stomach as visualized as yellow in the merged image. In the right of the lower panels showed that M4 receptors were not observed in the gastric mucosa of a M4 KO mouse.

To examine the presence of M4 receptors in D cells, we performed the immunostaining of the gastric mucosa with anti-somatostatin and anti-M4 receptor antibodies in WT mice. As expected, the expression of somatostatin was clearly observed in the stomach of WT mouse; it was stained red (**Figure 6**). On the other hand, the immunostaining of M4 receptors was also observed in the same area of the stomach; the higher magnification showed the co-existence of M4 receptors with somatostatin (a left figure of the lower panel), suggesting the expression of M4 receptors on D cells. No expression of M4 receptors was detected in the stomach of M4 KO mouse (a right figure of the lower panel), although the immunostaining of somatostatin was observed in this animal similar to WT mouse (data not shown).

### Changes in Serum Levels of Somatostatin after Administration of CCh in WT and M4 KO Mice

**FIGURE 7:**
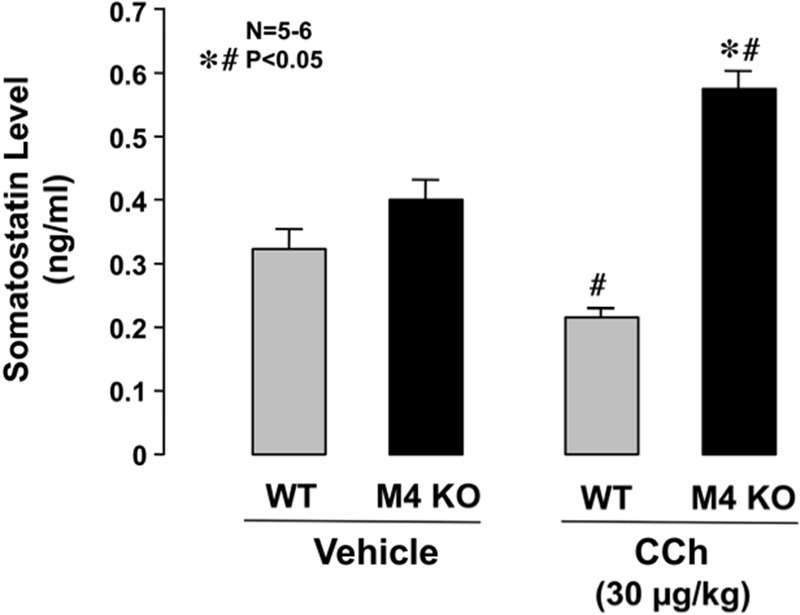
**Changes in serum somatostatin levels in WT or M4 KO mice after the administration of CCh.** CCh (30 μg/kg) was administered s.c. as a single injection. Data are presented as the means ± SE for 5 to 6 mice. Significant difference at *P* < 0.05; ^∗^ from WT; ^#^ from vehicle.

Serum levels of somatostatin in WT mice under urethane anesthesia were 0.33 ± 0.04 ng/ml. The levels of somatostatin in M4 KO mice were slightly higher (0.40 ± 0.04 ng/ml) than those in WT mice, although the difference was not statistically significant (**Figure 7**). On the other hand, the serum levels in WT mice were slightly but significantly decreased after the s.c. administration of CCh (30 μg/kg), the values being 0.22 ± 0.01 ng/ml. By contrast, the administration of CCh in M4 KO mice markedly increased the levels of somatostatin to 0.57 ± 0.04 ng/ml, the values being significantly higher than those in WT mice or those in M4 KO administered vehicle.

### Gene Expressions of mAChR Subtypes in Mouse Stomachs

**FIGURE 8:**
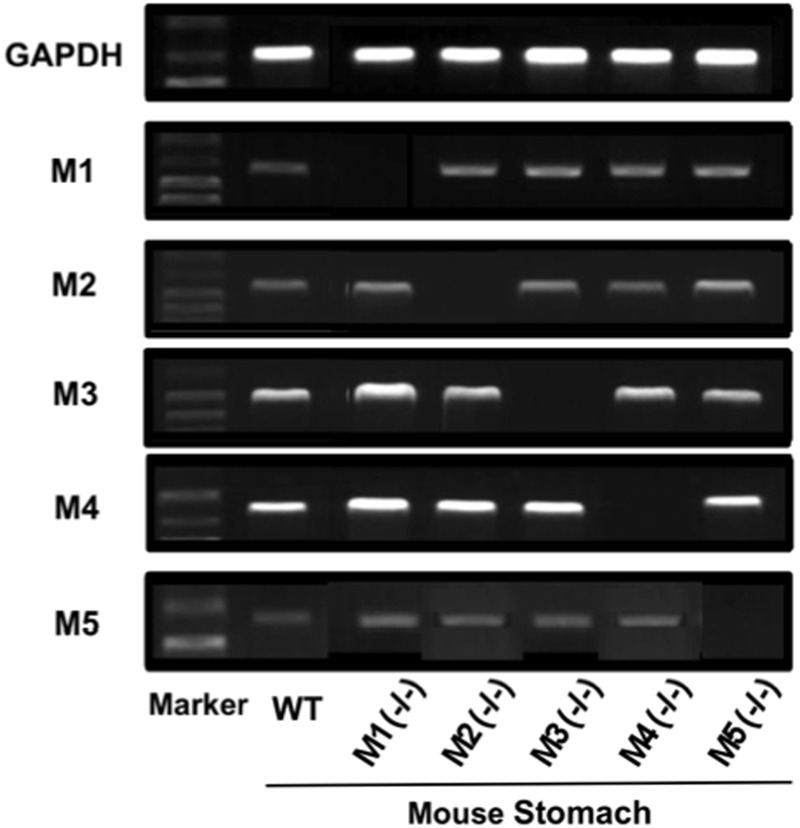
**Gene expressions of mAChR subtypes (M1–M5) in the mouse stomach.** Note that a respective mAChR subtype was lacking in the corresponding KO mice lacking M1–M5 receptors in the stomach.

Since it was found that both M3 and M4 receptors were involved in the stimulatory action of CCh on gastric acid secretion, we examined the gene expressions of mAChR subtypes, M1–M5, in the mouse stomach. The expressions of M1–M5 mRNAs were all observed in the stomachs of WT mice, though the intensity slightly differed depending on the subtypes (**Figure 8**). The expressions of mAChRs were also observed in the stomachs of KO mice lacking M1–M5 receptors, except lacking a respective mAChR subtype in the corresponding KO mice.

## Discussion

The parietal cell, which is responsible for gastric acid secretion, is known to express histamine H2 receptor and CCK2 receptors in addition to M3 receptors (Pfeiffer et al., 1990; Kajimura et al., 1992; Soll, 1994; Chen et al., 2004). The secretion of acid stimulated by Histamine or gastrin is mediated by the former two receptors, respectively (Brimblecombe et al., 1978; Soll, 1994; Chen et al., 2004), while the subtypes of mAChRs responsible for cholinergic stimulation of acid secretion remains fully unexplored (Nakamura et al., 1985; Pfeiffer et al., 1990; Matsui et al., 2004; Aihara et al., 2005). The mechanism of cholinergic stimulation of acid secretion has been thought to involve M1 and M3 receptors (Nakamura et al., 1985; Pfeiffer et al., 1990), yet a recent study reported that gastric acid secretion was normally stimulated by histamine and gastrin as well as CCh in M1 KO mice, suggesting that M1 receptors are not involved in the regulation of gastric acid secretion in mice (Aihara et al., 2005). In the present study, we demonstrated for the first time the involvement of muscarinic M4 receptors in the regulatory mechanism of cholinergic stimulation of gastric acid secretion.

The mAChRs, consisting of five subtypes (M1–M5), are widely expressed in many peripheral organs to include the gastrointestinal tract (Kajimura et al., 1992; Helander et al., 1996; Matsui et al., 2000; Tobin et al., 2009). In the present study, we confirmed by RT-PCR analysis that all mAChR subtypes including M1–M5 were expressed in the mouse stomach, although the degree of expression differed depending on the subtype; M3 and M4 receptors were potently expressed while M1, M2, and M5 were expressed weakly. In addition, we also demonstrated that the expressions of mAChRs were also observed in the stomachs of KO mice lacking M1–M5 receptors, except lacking a respective mAChR subtype in the corresponding KO mice. Others reported the expression of M1 receptors on zymogen cells and surface mucosal cells (Xie et al., 2005), that of M3 receptors on parietal cells (Pfeiffer et al., 1990; Kajimura et al., 1992; Aihara et al., 2005), and those of M2 and M4 receptors on D cell (Sachs et al., 1997; Takeuchi et al., 2015).

We found that CCh, a muscarinic agonist, dose-dependently increased acid secretion in the stomach of WT mice, and this response was almost completely inhibited by the prior administration of atropine, confirming the mediation of this secretion by the activation of mAChR. Cholinergic stimulation of gastric acid secretion is thought to involve M1 receptors in addition to M3 receptors, because acid secretion was inhibited by pirenzepine, a selective M1 receptor antagonist (Nakamura et al., 1985). However, Aihara et al. (2005) examined the involvement of M1, M3, and M5 receptors in cholinergic regulation of acid secretion using muscarinic receptor KO mice and found that CCh-stimulated acid secretion is mediated by mainly M3 and partially M5 but not M1 receptors. They also demonstrated that pirenzepine exhibited similar inhibitory effects on CCh-stimulated acid secretion in both WT and M1 KO mice, suggesting that inhibition of acid secretion by pirenzepine is unlikely to result from M1-receptor blockade (2005). On the other hand, M5 receptors might be expressed in the enteric nervous system and mediates cholinergic stimulation of acid secretion by increasing Ach release from nerve endings and/or releasing histamine from enterochromaffin-like cells (Aihara et al., 2005).

In the present study, we found using KO mice lacking M1–M5 receptors that CCh stimulated acid secretion in M1, M2, and M5 KO animals as effectively as in WT mice, but the stimulatory effect was markedly attenuated in M3, and M4 KO mice. We observed a slight decrease in the acid response to CCh in M5 KO mice, but the net acid output was not significantly different from that of WT mice. The reason for the different results between the study of Aihara et al. (2005) and our study, it may be due to different methods for acid measurement; they measure acid secretion in pylorus-ligated technique while we measured the secretion in the stomach with an acute fistula. Anyhow, it is therefore assumed that the cholinergic stimulation of gastric acid secretion is mediated by the activation of M3, and M4 receptors. In particular, the present study demonstrated for the first time the involvement of M4 receptors in the process of the CCh-stimulated gastric acid secretion, in addition to M3 receptors.

M3 receptors are coupled to Gq/11 protein to increase intracellular Ca^2+^ that mediates the secretion of acid as well as many hormones (Warhurst et al., 1996). Since acid secretion in response to CCh is known to be attenuated by a Ca^2+^ channel blocker, it is reasonable that M3 receptors are involved in the process of cholinergic stimulation of acid response (Pfeiffer et al., 1990; Aihara et al., 2005). It remains, however, unknown how the activation of M4 receptors modulates the acid response to CCh in the stomach. Since M4 receptors are coupled to Gi protein to decrease intracellular cAMP production (Caulfield and Birdsall, 1998), and since stimulation of gastric acid secretion is intracellularly mediated by cAMP, in addition to Ca^2+^ (Thurston et al., 1979; Soll, 1994; Hersey and Sachs, 1995), it is unlikely that M4 receptors are expressed in parietal cells and directly mediate the stimulation of acid secretion in response to CCh. Therefore, it is assumed that M4 receptors are expressed in cells other than parietal cells and indirectly affect the acid response to cholinergic stimulation.

By the way, somatostatin is synthesized in a variety of organs of the mammalian body and exerts almost ubiquitously an inhibitory action against various physiological processes (Lucey and Yamada, 1989). In the gastrointestinal tract, this peptide has been shown to inhibit motility and secretory functions and antagonizes the actions of several hormones (Lucey and Yamada, 1989; Warhurst et al., 1996; Terashima et al., 2009). Chiba and Yamada (1990) reported that CCh inhibited both basal and pentagastrin- stimulated somatostatin secretion in a Gi protein/cAMP-dependent manner in the isolated canine D cells. Five subtypes, SST1-SST5, of somatostatin receptors are currently known to exist, and all of them are expressed in the gastrointestinal tract (Chiba and Yamada, 1990; Krempels et al., 1997; Patel, 1997). It is thus possible that the activation of M4 receptors inhibits somatostatin secretion from D cells and by so doing indirectly affect acid secretion. If this is the case, the followings should be demonstrated; (i) somatostatin suppresses the acid response to cholinergic stimulation such as CCh, (ii) the decreased acid response in M4 KO mice can be reversed by the SST2 antagonist, and (iii) the release of somatostatin from D cells is increased in M4 KO mice. As expected, we found that CCh-stimulated acid secretion was significantly suppressed by octreotide, the somatostatin analog. We also found that the suppressed acid response to CCh in M4 KO mice was significantly restored by the prior application of the somatostatin SST2 antagonist, CYN154806. In addition, it was found that serum somatostatin levels were significantly increased in M4 KO mice under CCh-stimulated conditions. These results strongly support our hypothesis that the decrease of CCh-induced acid response in M4 KO mice is explained by the inhibitory effect of somatostatin mediated by SST2 receptors. It is therefore assumed that the activation of M4 receptors inhibits somatostatin release from D cells and negates the negative influence of this peptide on acid secretion, resulting in a potentiation of the acid response to CCh. Certainly, more studies are needed to clarify the regulatory mechanisms of somatostatin secretion from D cells.

Finally, it remains undefined whether M4 receptors are really expressed on D cells? We performed the immunostaining of the gastric mucosa with anti-somatostatin and anti-M4 receptor antibodies in WT mice. The histological observation showed that M4 receptors were co-expressed with somatostatin, indicating the expression of M4 receptors on D cells. We confirmed that M4 receptors were not observed in the stomachs of M4 KO mice. These results strongly suggest that CCh inhibits somatostatin release from D cells via the activation of M4 receptors. The present study was performed in mice anesthetized with urethane. Since this anesthetic is known to promote the secretion of somatostatin from D cells (Saito et al., 1979), the results obtained in this study might differ from those obtained under normal physiological conditions. However, since CYN154806 by itself had no effect on basal acid secretion, it is assumed that the interpretation of the present results is not affected by urethane anesthesia.

**FIGURE 9:**
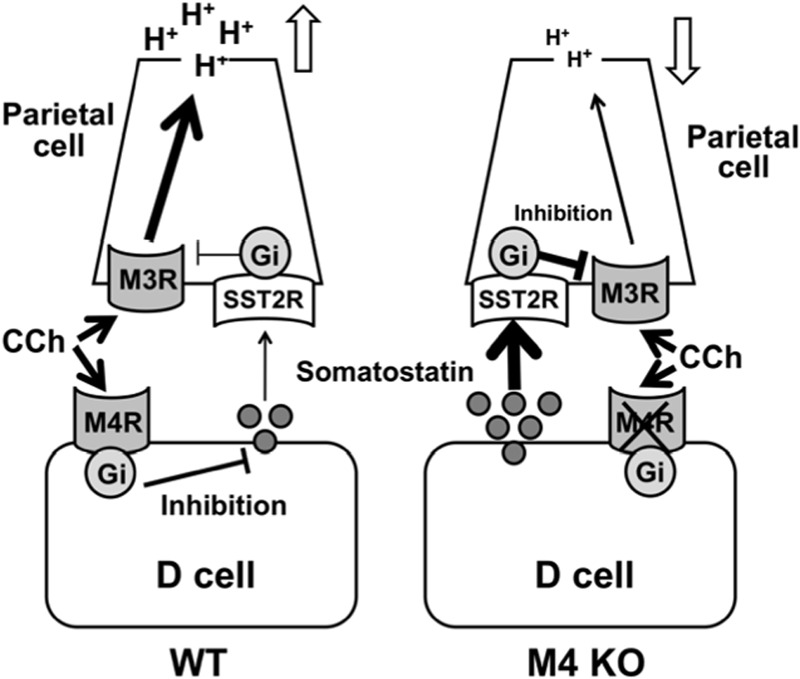
**Working hypothesis for the involvement of M4 receptors in the regulatory mechanism of cholinergic stimulation of gastric acid secretion.** The cholinergic stimulation of acid secretion is mediated by the activation of M3 and M4 receptors. The activation of M3 receptors directly affects acid secretion from the parietal cells through a Gq protein/Ca^2+^ pathway, while the activation of M4 receptors inhibits somatostatin secretion from D cells and by so doing unmasks the stimulatory effect of CCh mediated by the activation of M3 receptors in the parietal cells.

Given the findings of the present study, we conclude that the cholinergic stimulation of gastric acid secretion is mediated by the activation of M3 receptors in parietal cells and modified indirectly by M4 receptors in D cells (**Figure 9**). It is assumed that the activation of M4 receptors inhibits the release of somatostatin from D cells to result in enhancement of the acid response by removing the negative influence of somatostatin via the activation of SST2 receptors.

## Author Contributions

KT authored the paper and designed the study; TE, SH, and TA performed the experiments; TE and SH performed data analysis and coauthored the paper; KT contributed to critical revision of the paper. All authors approved the submission of the manuscript.

## Conflict of Interest Statement

The authors declare that the research was conducted in the absence of any commercial or financial relationships that could be construed as a potential conflict of interest.
